# Depressive scores in newly diagnosed HIV-infected and HIV-uninfected pregnant women

**DOI:** 10.4102/sajpsychiatry.v23i0.1085

**Published:** 2017-12-01

**Authors:** Puvashnee Nydoo, Thajasvarie Naicker, Jagidesa Moodley

**Affiliations:** 1 Women’s Health and HIV Research Unit, Nelson R Mandela School of Medicine, University of KwaZulu-Natal, South Africa; 2 Optics and Imaging Centre, Nelson R Mandela School of Medicine, University of KwaZulu-Natal, South Africa

## Abstract

**Background:**

Prevalence rates of HIV infection in KwaZulu-Natal are high, with a significant amount of those infected being women of reproductive age. A diagnosis of HIV infection has been associated with an increased risk for the development of depression. Antenatal depression is a serious health concern, having the potential to cause wide-reaching adverse consequences for mother and unborn child.

**Aim:**

To compare depressive scores between newly diagnosed HIV-infected and HIV-uninfected pregnant women.

**Setting:**

Antenatal clinics at two regional hospitals in KwaZulu-Natal, South Africa.

**Methods:**

A cross-sectional questionnaire-based analysis of 102 newly HIV-tested black African pregnant women (HIV infected: *n* = 40; HIV uninfected: *n* = 62) was conducted. Women’s socio-demographic and clinical data were recorded, before being assessed for depressive symptomology using an isiZulu version of the Edinburgh Depression Scale.

**Results:**

About 9.8% of women suffered from significant depressive symptoms, irrespective of HIV status. Prevalence rates of antenatal depressive symptoms did not differ significantly between HIV-infected and HIV-uninfected cohorts (*p* = 0.79). A new diagnosis of HIV infection (*p* < 0.0001) and maternal age (*p* = 0.03) were risk factors for antenatal depression. Unemployment was a borderline risk factor (*p* = 0.09) for the development of antenatal depression.

**Conclusion:**

Prevalence rates of depressive symptoms were low. Knowledge of a new diagnosis of HIV infection at the first antenatal visit places women at an increased risk for the development of depression during pregnancy. Younger age and unemployment influence depression. This study provides an important step in documenting the need for screening for antenatal depression in HIV-associated pregnancies in a South African population group.

## Introduction

Women represent approximately half (51.0%) of adults living with HIV infection worldwide,^1^ with the majority of infections occurring in women of reproductive age. Furthermore, the majority of women living with HIV globally are from sub-Saharan Africa, and an estimated 1.5 million of these women become pregnant each year.^2^ In South Africa, one-fifth of women in their reproductive age are HIV infected, and recent HIV prevalence rates indicate that up to 22.8% of pregnant women are HIV infected.^3^ In the province of KwaZulu-Natal (KZN) alone, the antenatal rate of HIV infection is approximately 30.0%.^4^

A diagnosis of HIV infection is associated with a surge in depressive symptoms.^5,6^ According to WHO Department of Health Statistics and Informatics, depressive disorders account for nearly half of the burden of disease exhibited by mental disorders.^7^ In South Africa alone, it is estimated that 9.8% of adults will experience a major depressive episode (MDE) at least once in their lifetime.^8^ Additionally, research has shown that rates of depression are higher in women than men, further highlighting the fact that pregnancy is not always a time of emotional well-being and depression during pregnancy is common, with prospective studies reporting similar rates of depression between pregnant and non-pregnant women.^9^ A recent study conducted amongst a South African population indicates that more than a third of women have significant depression during pregnancy.^6^ Antenatal depression is a considerable health concern, having the potential to compromise the well-being of both mother and infant.^5^ Adverse outcomes include obstetric complications such as miscarriage, preterm labour, low birth weight babies and foetal growth restriction, as well a negative effect on the cognitive functioning of the mother.^6^

A diagnosis of HIV infection is accompanied by psychiatric distress, with depression being a common complication.^10^ Studies have found that depression is highly prevalent in an HIV-infected population, with HIV-infected individuals being almost twice as likely to be depressed when compared to HIV-uninfected individuals.^11,12^ A new HIV diagnosis increases this risk of depression in pregnancy.^13^ Prevalence rates of antenatal depression in South Africa are high, with rates of 38.5% in urban KZN, 39% in Cape Town, 41.0% – 47.0% in rural KZN and 48.7% in Mpumalanga.^6,14,15,16,17^ Depression in HIV-infected pregnant women can negatively impact the adherence of antenatal regimes, which may aggravate disease progression,^18^ invariably increasing the risk of adverse maternal and infant outcomes.

The knowledge and timing of this diagnosis can also affect the development of depression. Studies found that women who are recently diagnosed with HIV infection are at a higher risk of depression, whereas women who know their HIV status before becoming pregnant were less likely to develop depressive symptoms.^13^ These symptoms were attributed to the relatively short time period for adjustment to the diagnosis, the stigma attached to being pregnant whilst HIV infected,^5^ as well as the fear of placing the unborn child at risk of HIV infection.^6^

However, it is controversial whether HIV infection impacts the frequency of depressive rates in pregnant women. As KZN is considered to be the global epicentre of the HIV pandemic,^4^ its high prevalence rates of HIV in pregnancy and the likelihood of the burden of antenatal depression, we are well positioned to study the trio of HIV infection, depression and pregnancy. Thus, the aim of this study is to compare depressive scores between newly diagnosed HIV-infected and HIV-uninfected pregnant women using the Edinburgh Depression Scale (EDS) in KZN to elucidate any association between a new diagnosis of HIV infection and the development of antenatal depression.

## Research methods and design

### Study design

A cross-sectional questionnaire-based analysis of depressive scores in newly HIV-tested pregnant women was conducted.

### Study site and study population

From August 2016 to October 2016, a sample of 102 pregnant women (62 HIV-uninfected and 40 HIV-infected) were recruited from antenatal clinics at two regional hospitals in KZN, South Africa. Patients were recruited based on the study’s inclusion and exclusion criteria. Given the study design, only newly HIV-tested women (prior to initiation of antiretroviral therapy) were included. Only Black South African women were included as there is a higher percentage of this population group attending these hospitals than other demographic groups. Additional inclusion criteria were being isiZulu speaking and period of gestation (either second or third trimester). Pregnant women previously tested for HIV, non-black South African patients and those that declined entry into the study were excluded. Furthermore, patients in the first trimester of pregnancy, those with medical and surgical complications, as well as individuals with a previous history of psychiatric illness, were excluded.

### Data collection

Whilst women waited for their routine antenatal appointment, the purpose and nature of the study was explained to the whole group by the research nurse. Research participants were then given individual patient information sheets in isiZulu and invited to participate. Socio-demographic and clinical data including maternal age, gestational age, parity, whether the pregnancy was planned or unplanned, area of residence, level of education, employment status, substance use, relationship status, HIV status and CD4 cell counts were recorded on a structured data sheet and participants were then assessed for depressive symptomology using the EDS. This 10-item self-report scale relates to an individual’s depressive symptoms during the past 7 days. Each item is scored on a 4-point scale with a total score range of 0–30, where a higher score indicates greater distress. It focuses on the individuals’ cognitive and affective depressive symptoms, whilst omitting somatic symptoms that could be confounded by normal pregnancy-related changes. It was originally established in 1987 and named the Edinburgh Postnatal Depression Scale, as it was developed as a screening tool for postpartum depression.^19^ However, this scale is now also validated as a screening tool for depression during pregnancy,^20^ giving rise to a new nomenclature: the EDS. It is an easily administered questionnaire and has been previously validated in antenatal Black South African women,^6^ specifically amongst newly diagnosed HIV-infected antenatal women in South Africa.^16^ The EDS was translated into an isiZulu version for the purpose of this study and then completed in the form of interviews by each participant with the assistance of a Zulu-speaking research nurse. Based on previous studies, interpretation of scores was as follows: scores 0–9 may indicate the presence of some short-term symptoms of distress that are not likely to interfere with the individual’s ability to function at home or at work on a daily basis, scores 10–12 indicate the presence of symptoms of distress that may be discomforting and scores of ≥ 13 require further evaluation and possible referral to a perinatal mental health specialist. For the purpose of this study, we used these cut-off scores to determine our three depressive severity symptom groups (0–9: no depression, 10–12: significant distress and ≥ 13: probable depression). Given the cut-off score for likely depression, women who scored ≥ 13 were offered referrals to the psychiatric clinic at either hospital.

### Data analysis

Data were analysed using GraphPad Prism 5.00 for Windows (GraphPad Software, San Diego, California, USA) and STATA version 12 (StataCorp LP, College Station, Texas, USA). Parametric tests were performed. Continuous variables were described in terms of means ± standard deviations and categorical variables using their frequencies and percentages. *T*-tests were generated to examine the associations between categorical variables and depressive scores. Spearman’s correlation coefficients were generated to determine the association between continuous variables and depressive scores. Frequency distributions were also calculated. Statistical significance was determined by a *p*-value of < 0.05.

## Ethical consideration

Prior to the commencement of study activities, ethical approval (BE271/16) and permission to conduct the study at a large district and a tertiary referral hospital, was obtained. All participants signed a written informed consent form and were ensured confidentiality and anonymity through the use of research codes

## Results

### Patient socio-demographic data

Background characteristics of study population are depicted in Table 1. Maternal age of the sample ranged from 17 to 40 years, with a mean ± standard deviation of 25 ± 5.30 years. Gestational age of the sample had a mean ± standard deviation of 18+ weeks ± 5.52 (18 ± 5.31 for HIV-infected versus 20 ± 5.75 for HIV-uninfected). More than two-thirds of the participants had completed secondary school education (62.5% HIV-infected vs. 69.4% HIV-uninfected); unemployment rate was high across the sample, with 70.0% for HIV-infected versus 80.6% for HIV-uninfected women. The majority of women were single (90.0% HIV-infected vs. 93.6% HIV-uninfected), whereas 80.0% of HIV-infected and 87.1% of HIV-uninfected women had not planned to fall pregnant. Of the 102 women, 14.7% reported having miscarriages in previous pregnancies, with more cases reported in the HIV-uninfected cohort (16.1%). The mean CD4 count for the HIV-infected cohort was 425 cell/mm^3^.

**TABLE 1:** Background characteristics of study population.

Characteristics	HIV-infected (*n* = 40) *n* (%) or mean ± s.d.	HIV-uninfected (*n* = 62) *n* (%) or mean ± s.d.	*p*
Age (years)	28 ± 4.86	22 ± 2.83	0.0001
**Planned pregnancy (%)**			0.34
Yes	8 (20)	8 (12.90)	
No	32 (80)	54 (87.10)	
**Area of residence (%)**			0.07
Urban	40 (100)	57 (91.94)	
Rural		5 (8.06)	
**Highest level of education (%)**			0.35
No formal education	1 (2.5)	0(0)	
Primary school	1 (2.5)	0(0)	
Secondary school	25 (62.5)	43 (69.4)	
University	13 (32.5)	19 (30.6)	
**Employment status (%)**			0.22
Employed	12 (30)	12 (19.4)	
Unemployed	28 (70)	50 (80.6)	
**Relationship status (%)**			0.52
Single	36 (90)	58 (93.6)	
Married	4 (10)	4 (6.4)	

Age is presented as mean ± s.d. (standard deviations). Planned pregnancy, area of residence, level of education, employment status and relationship status are presented as frequencies (*n*) and percentages (%).

### Prevalence and correlates of depression

Associations between socio-demographic and clinical factors and total depressive scores on the EDS are represented in Table 2. Evaluation of the EDS demonstrated a Cronbach’s alpha of 0.76.

**TABLE 2:** Associations between socio-demographic and clinical factors and total depressive scores on the Edinburgh Depression Scale.

Characteristics	Total depressive scores mean ± s.d.	*p*
**HIV status**		0.0001*
Infected	10.43 ± 3.46	
Uninfected	6.77 ± 3.13	
**Planned pregnancy**		0.87
Yes	8.06 ± 4.60	
No	8.23 ± 3.55	
**Highest level of education**		0.55
Secondary school	8.06 ± 3.74	
University	8.53 ± 3.67	
**Employment status**		0.09
Employed	8.55 ± 3.34	
Unemployed	7.08 ± 4.61	
**Relationship status**		0.65
Single	8.26 ± 3.65	
Married	7.63 ± 4.53	

s.d., standard deviation.

* , Result significant at *p* = 0.0001

**FIGURE 1:**
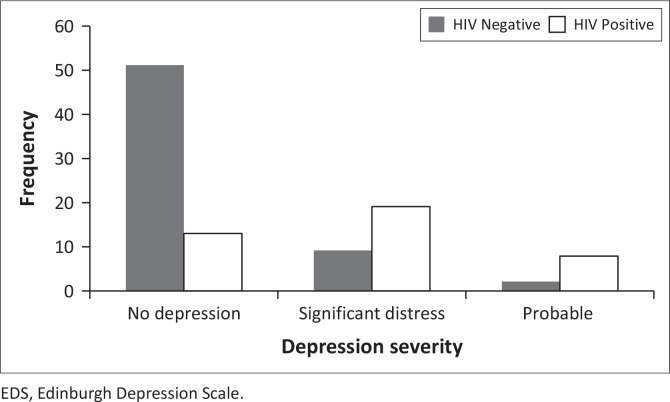
Prevalence of depressive symptoms amongst 102 newly diagnosed HIV-infected and HIV-uninfected pregnant women in KZN, South Africa. No depression: EDS scores of 0–9; significant distress: EDS scores of 10–12; probable depression: EDS scores of 13–30.

As shown in Figure 1, of the sample of 102 women, 10 (9.8%) had an EDS score of ≥ 13, indicating probable depression. The majority of the women exhibiting symptoms of probable depression fell within the HIV-infected cohort (7.8%). Amongst the HIV-infected cohort, 13% of women displayed symptoms of probable depression, whereas only 5.0% of women in the HIV-uninfected cohort presented symptoms of probable depression. Additionally, 18.6% of HIV-infected women displayed symptoms of significant distress. No depression was reported by 50.0% of HIV-uninfected women. We report no significance between HIV-infected (*M* = 7.08, s.d. = 1.56, *n* = 13, 95.0% CI: 6.14–8.02) and HIV-uninfected (*M* = 5.82, s.d. = 2.39, *n* = 51, 95.0% CI: 5.15–6.50) women across the no depression group; no significance between HIV-infected (*M* = 10.37, s.d. = 0.60, *n* = 19, 95.0% CI: 10.08–10.66) and HIV-uninfected (*M* = 10.22, s.d. = 0.44, *n* = 9, 95.0% CI: 9.88–10.56) women across the significant distress group; and no significance between HIV-infected (*M* = 16, s.d. = 2.33, *n* = 8, 95.0% CI: 14.05–17.95) and HIV-uninfected (*M* = 15.50, s.d. = 2.12, *n* = 2, 95.0% CI: -3.56–34.56) women across probable depression group (*p* > 0.05).

**FIGURE 2:**
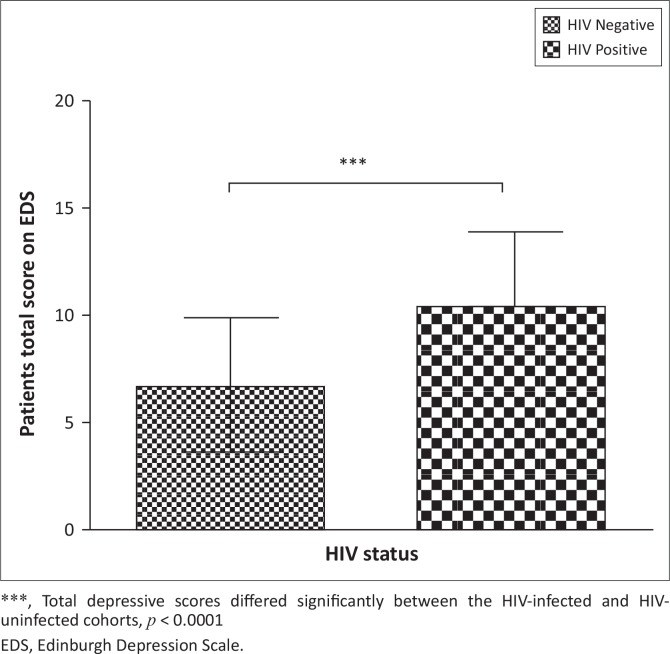
Depressive scores of HIV-infected versus HIV-uninfected pregnant women. Results are presented as mean ± standard deviations.

**FIGURE 3:**
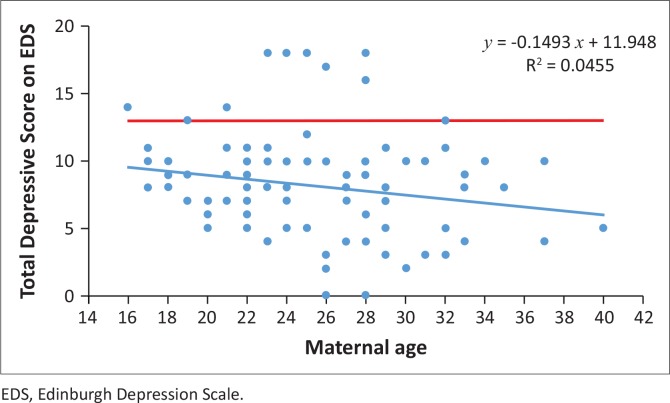
Correlation between maternal age and total depressive scores on the EDS. The red line represents the cut-off score for probable depression (13) on the EDS. *, Total depressive scores differed significantly with maternal age of women, *p* = 0.03.

Analysis amongst women’s total depressive scores from the EDS, irrespective of depressive severity, showed a significant association between depression and HIV status and maternal age, which are further highlighted in Figures 2 and 3, respectively. The total depressive scores differed significantly between HIV-infected (*M* = 10.43, s.d. = 3.46, *n* = 40, 95% CI: 9.317393–11.53261) and the HIV-uninfected (*M* = 6.77, s.d. = 3.13, *n* = 62, 95% CI: 5.979836–7.568551) women (*p* < 0.0001). HIV-infected women were more prone to higher scores on the EDS than HIV-uninfected women (*t* = -5.52, df = 100). Total depressive scores differed significantly with maternal age of women at the 0.05 level of significance, indicating that younger women were more inclined to higher scores of depressive symptoms (*r* = 0.21; *p* = 0.03). There were no significant correlations between level of education (*p* = 0.55), whether pregnancy was planned or not (*p* = 0.87), relationship status (*p* = 0.65), CD4 cell count (*p* = 0.69) and severity of depressive scores. There was a trend association between employment status and total depressive scores (*p* = 0.09). Unemployed women (*M* = 8.55; s.d. = 3.34; *n* = 78; 95% CI: 7.80–9.30) were more depressed than employed women (*M* = 7.08; s.d. = 4.61; *n* = 24; 95% CI: 5.13–9.03).

## Discussion

### Key findings

A new diagnosis of HIV infection during pregnancy can affect the severity of depressive scores. In this study, we used the EDS to evaluate the prevalence and severity of depressive scores in newly diagnosed HIV-infected and HIV-uninfected pregnant women in order to elucidate the role of a new diagnosis of HIV infection in the development of depression.

We report a low prevalence rate of depressive symptomology regardless of HIV status across the study population. Notably, the majority of women displaying scores consistent with significant depressive symptoms were from the HIV-infected cohort. However, when only considering women with probable depression (equivalent to a score of ≥ 13), no significant difference in the prevalence of depressive symptoms between the HIV-infected compared to the HIV-uninfected cohorts was found (*p* = 0.79).

Moreover, our study reports that women’s total depressive scores, irrespective of depressive severity, were significantly different between HIV-infected and HIV-uninfected pregnant women. Higher depressive symptom scores were demonstrated in the HIV-infected cohort, highlighting that a new diagnosis of HIV infection predisposes a pregnant woman to a greater risk of developing depression. Interestingly, amongst the HIV-infected cohort of our study, the majority of women displayed symptoms of significant distress rather than that of probable or severe depression. This was also highlighted in other studies which found a diagnosis of HIV infection to increase the risk and severity of emotional distress in pregnant women.^12^

A significant difference in maternal age was noted between the study cohorts. HIV-infected pregnant women were much older than HIV-uninfected pregnant women. Additionally, we found that as maternal age increased, the level of significant depressive symptoms decreased, demonstrating that younger women were more inclined to develop depression.

Also as expected, unemployed pregnant women were more depressed than employed women. Although only borderline significant (*p* = 0.09), an unemployed status was noted to have a minor effect on severity of depressive scores.

### Discussion of key findings

Our study demonstrates that 9.8% of pregnant women displayed symptoms of depression, irrespective of HIV status. This rate of depressive symptoms is lower than expected and contradicts previous literature in which higher prevalence rates of depression were found in similar settings: 38.5% in urban KZN and 47.0% in rural KZN.^6,16^ Our low rate of depressive symptoms may be attributed to South Africa’s shift to a low-middle income country, in which prevalence of antenatal depression (15.6%) is similar to our study.^21^ This was corroborated by the fact that 95.0% of women in our sample resided in urban areas and that the majority of women had a moderate-high level of education. Furthermore, the gestational periods at which women were recruited differed with previous studies, which could further explain our low prevalence of depressive symptomology.^13,22^

We report that total depressive scores on the EDS, irrespective of depressive severity, differed significantly between the HIV-infected and HIV-uninfected cohort. This finding is not novel, and evidence of elevated depressive scores amongst HIV-infected pregnant women has been previously reported.^6^ It is possible that in our group, because we interviewed only newly diagnosed pregnant women, these results may be attributed to an adjustment reaction to the recent report of a positive HIV diagnosis. Analogous evidence supporting this hypothesis has been noted previously.^5^ Nevertheless, our finding suggests that a new diagnosis of HIV infection in pregnancy increases the development of depressive symptoms.

However, we did not find a difference in prevalence rates of depressive symptoms between the HIV-infected and HIV-uninfected cohorts. Similar findings were reported in HIV-infected and HIV-uninfected pregnant women in South Africa and the US.^16,23^ Yet, our finding contradicts other reports, in which prevalence of depression was significantly greater amongst HIV-infected pregnant women.^6,13^ Our finding might be explained by the province’s high HIV prevalence rate, which may have destigmatised the disease, thus making a diagnosis of HIV infection more acceptable. Additionally, accessibility to antiretroviral therapy and medical regimes has improved in South Africa and has led to decreased mortality by slowing the progression of HIV to full-blown AIDS; thus, shifting the status of HIV infection from a death sentence to a chronic illness may also be a factor explaining these findings. Similar correlations are also evident in other studies, in which the idea of HIV infection as a chronic disease has made a diagnosis increasingly normalised.^24^

Maternal age is a predictor of depression, indicating that younger women are at a greater risk of developing depression than older women.^22^ Our results corroborate these findings, in that younger maternal age was associated with a higher level of depressive symptoms. The analogous rates of depressive symptoms between both cohorts may be further explained by the increased maternal age of the HIV-infected cohort.

Unemployed women were at a greater risk of developing depression than employed women in this study. This may be elucidated by the increased financial and social support one receives when having a permanent job. This finding has been demonstrated previously^21^ and is evidence that socio-economic circumstances contribute to the development and severity of depressive symptoms.

In contrast to previous reports, we were unable to demonstrate correlations between level of education, planned pregnancy, relationship status and CD4 cell counts.^6,25^ With regard to education and relationship status, failure to attain any significant difference in our study may be because of the unequal sample size between the study cohorts and the majority of women falling within the secondary school and single categories, respectively. Although other studies have found lower CD4 cell counts to be associated with higher rates of depression,^25^ our study did not find any difference in depressive symptomology rates according to CD4 cell count.

### Strengths and limitations

This study reports and compares depressive scores of newly diagnosed HIV-infected and HIV-uninfected pregnant women in KZN, South Africa. It provides valuable information on the prevalence, as well as the role played by HIV infection and other socio-demographic and clinical data as risk factors for the development of depressive symptoms in pregnant women, in a province that is the foremost contributor to the global HIV pandemic. The EDS also demonstrated good internal reliability, with a Cronbach’s alpha of 0.76, indicating that women in this study were consistent in their response.

Our study has limitations. One is the relatively small sample size, accompanied by the unequal sample size between cohorts. The study population was limited to Black African women; therefore, it is not known whether the results can be generalisable to other populations. Additionally, this study did not examine antenatal depression across all three trimesters of pregnancy, which could possibly impact the prevalence rate. It is also possible that women were not truly ‘newly diagnosed’, which could have affected the prevalence rate in this study. Furthermore, certain variables noted in previous studies that were found to be associated with depressive symptoms were not assessed in this study. Finally, the cross-sectional design of the study limits us to mere associations and prevents actual causal inferences.

### Recommendations

Given the likelihood of the burden of antenatal depression on the quality of life of both mother and infant, future studies conducting research using a clinical diagnostic instrument for depression on a larger sample, involving all three trimesters of pregnancy and following delivery, are recommended.

## Conclusion

We report a low prevalence of significant depressive symptoms in pregnancy, irrespective of HIV status, which may be attributed to the improved perception of HIV infection in South Africa. Additionally, we report that a new diagnosis of HIV infection in pregnancy exacerbates the risk for the development of depressive symptomology. Moreover, we demonstrate that younger maternal age and unemployment increase the development of depressive symptoms. Furthermore, we report similar rates of depressive symptoms between HIV-infected and HIV-uninfected cohorts. This study provides an important step in documenting the need for screening for antenatal depression across all three trimesters and following delivery in HIV-associated pregnancies.
